# Key Parameters Impacting the Crystal Formation in Antisolvent Membrane-Assisted Crystallization

**DOI:** 10.3390/membranes13020140

**Published:** 2023-01-21

**Authors:** Sara Chergaoui, Damien P. Debecker, Tom Leyssens, Patricia Luis

**Affiliations:** 1 Institute of Mechanics, Materials and Civil Engineering—Materials & Process Engineering (iMMC-IMAP), Université Catholique de Louvain (UCLouvain), Place Sainte Barbe 2, 1348 Louvain-la-Neuve, Belgium; 2 Research & Innovation Centre for Process Engineering (ReCIPE), Université Catholique de Louvain (UCLouvain), Place Sainte Barbe, 2 bte L5.02.02-B, 1348 Louvain-la-Neuve, Belgium; 3 Institute of Condensed Matter and Nanosciences (IMCN), Université Catholique de Louvain (UCLouvain), Place Louis Pasteur, 1 bte L4.01.06, 1348 Louvain-la-Neuve, Belgium

**Keywords:** antisolvent crystallization, hydrophobic membrane, velocity, temperature, antisolvent composition, gravity

## Abstract

Antisolvent crystallization is commonly used in the formation of heat-sensitive compounds as it is the case for most active pharmaceutical ingredients. Membranes have the ability to control the antisolvent mass transfer to the reaction medium, providing excellent mixing that inhibits the formation of local supersaturations responsible for the undesired properties of the resulting crystals. Still, optimization of the operating conditions is required. This work investigates the impact of solution velocity, the effect of antisolvent composition, the temperature and gravity, using glycine-water-ethanol as a model crystallization system, and polypropylene flat sheet membranes. Results proved that in any condition, membranes were consistent in providing a narrow crystal size distribution (CSD) with coefficient of variation (CV) in the range of 0.5–0.6 as opposed to 0.7 obtained by batch and drop-by-drop crystallization. The prism-like shape of glycine crystals was maintained as well, but slightly altered when operating at a temperature of 35 °C with the appearance of smoother crystal edges. Finally, the mean crystal size was within 23 to 40 µm and did not necessarily follow a clear correlation with the solution velocities or antisolvent composition, but increased with the application of higher temperature or gravity resistance. Besides, the monoclinic form of α-glycine was perfectly maintained in all conditions. The results at each condition correlated directly with the antisolvent transmembrane flux that ranged between 0.0002 and 0.001 kg/m^2^. s. In conclusion, membrane antisolvent crystallization is a robust solution offering consistent crystal properties under optimal operating conditions.

## 1. Introduction

Membranes have been serving us well in different sectors such as water purification, gas separation and CO_2_ capture [1,2,3,4], but novel applications are emerging nowadays. Membrane-assisted antisolvent crystallization (MAAC) is a new technique to control antisolvent crystallization. The latter consists of adding an antisolvent to a solvent-solute mixture, so that the solute crystallizes when reaching supersaturation [5]. In MAAC, the membrane acts as a physical barrier between the two solutions: the crystallizing solution which contains the solvent and solute to be crystallized, and the antisolvent solution, meaning a solution that is miscible to the solvent but does not dissolve the solute. This way, the antisolvent gradually decreases the solubility of the solute to the point where supersaturation is reached, and crystals are formed [6]. 

A conventional antisolvent crystallizer in pharma- or agro-industries consist of a large batch container of the feed solution, mixed with the antisolvent where crystals emerge the minute antisolvent is added [7]. This quick crystallization lacks control over the resulting crystal size, crystal morphology and sometimes crystalline system in the sense that a tertiary polymorph may appear in the solid mixture [8,9,10]. The solution is to adopt a process unit that gives well-distributed crystals from liquid solution, without compromising the crystal quality. Besides the control of mixing and antisolvent mass transfer, the incorporation of the membrane into the crystallization system results in the confinement of the solution in a nano/micrometer scale, that redefines the free energy of crystal surface, structure and properties [11]. MAAC has as well the potential to be used as a technology in the continuous manufacturing of chemical products, which can potentially reduce the complexity of the system, the capital investment and operating cost of downstream processing is reduced [12].

The potential applications of MAAC are wide. MAAC can be used for example to control polymorphism and co-crystallization [13,14,15]. Di Profio et al. [16] used solvent/antisolvent demixing configuration where the membrane represented a barrier between two solutions such that solvent exchange between either side of the membrane decreased the solubility of carbamazepine (an active pharmaceutical ingredient) with the co-former saccharin. MAAC can also possibly be used in medical investigations: It would be interesting to simulate gastrointestinal drug supersaturation, or how kidney stones form [17]. MAAC can intensify polymer coating of nanoparticles. Chen et al. used hollow fiber membranes to continuously coat submicron and nanosized silica particles with a thin polymer layer. A suspension of the particles in an acetone solution of the polymer was continuously pumped through the shell-side of a hollow fiber membrane module, while water (the antisolvent) was flowing in the hollow fiber lumen side, and was forced through the membrane pores into the shell side, generating intense mixing with the flowing suspension and rapid coating of the particles [18].

Polymeric membranes adequate for MAAC operation are commonly made of polypropylene (PP), polyvinylidene fluoride (PVDF) or polytetrafluoroethylene (PTFE) among others [19]. The hydrophobicity is necessary to avoid membrane wetting by the crystalizing solution, while the pores ensure the transfer of antisolvent due to activity/concentration difference between the two sides of the membrane. Besides the adequate membrane type, the optimum operating conditions are equally necessary. Previous studies (Table 1) have reported different conditions during MAAC operation. Mostly, the velocity of either the crystallizing or antisolvent velocity, was optimized [20,21,22,23], other studies preferred varying the antisolvent concentration or the temperature [24]. Besides, the membrane module position, i.e., whether placed horizontally or at a different angle, could affect the gravity resistance, hence the flowability of the solution and avoid clogging with the formed crystals [23]. Nonetheless, a correlation between these parameters and the resulting crystal properties has not yet been established, so this study investigates the impact of these key four parameters, i.e., the solution velocity, the antisolvent composition, the temperature and gravity resistance on the crystal properties (i.e., the crystal shape, crystalline system, and size distribution). The impact of additional pressure was deemed unnecessary as it results in a high transmembrane flux of the antisolvent which nullifies the essential role of membrane in controlling the mass transfer. 

Glycine is a particularly preferred model compound since it is an amino acid with a simplistic molecular structure. The glycine molecule contains an amino group –NH_2_ and a carboxylic acid group –COOH and exists as zwitterions in both crystals and solutions with ammonium and carboxylate ions at its two ends [25,26,27]. From an industrial perspective, glycine has potential applications in the pharmaceutical industry as an active pharmaceutical ingredient and exhibits interesting nonlinear optical properties. In this study, glycine is the solute, water is the solvent and ethanol or a mixture of ethanol/water is used as the antisolvent. This specific antisolvent crystallization system represents typical antisolvent crystallization systems, placing this work in the wider context of antisolvent particle formation processes. 

**Table 1:** Operation conditions that figured in previous MAAC studies, plus the main results regarding antisolvent mass transfer and crystal properties. * [A] Antisolvent; [CS] Crystallizing solution.

Crystallization System *	Solution Velocity		Antisolvent Composition	Temperature [K]	Gravity	Key Results	Ref.
	Crystallizing Solution	Antisolvent Solution					
[A] Water and ethanol mixture at 30 C; [CS] Glycine solution in bi-distilled water at 10 C.	-	-	40% ethanol in ethanol/water mixture	Antisolvent and crystallizing solution were set at 303.15 and 283.15 K respectively.	-	The transmembrane flux was J = 1.53 or 9.1 × 10^−4^ Lh^−1^.	[24]
[A] Ethanol; [CS] A solution of erythritol and ultrapure water.	10 to 20 mL·min^−1^	60 to 120 mL·min^−1^	100% ethanol	293.15	From the schematic, the hollow fiber module was perhaps placed vertically.	The transmembrane flux ranged between 2 and 4 Kg·m^−2^·h^−1^; CV was 48%.	[20]
[A] Ethanol; [CS] A solution of NaCl and ultrapure water	93 mL·min^−1^	10 mL·min^−1^	100% ethanol	Ambient temperature, which in China can be 303.15	The hollow fiber module was perhaps placed vertically.	CV was 43.5%	[28]
[A] Deionized water; [CS] Indomethacin in ethanol.	10–15 and 20 mL·min^−1^	25 mL·min^−1^	100% water	-	The hollow fiber module was placed horizontally.	Average velocity across membrane was 1.2–1.4 m/s Particle median diameter was D_50_ = 0.3 to 0.35 µm.	[21]
[A] Deionized water; [CS] Drug particles of Griseofulvin in acetone.	6–12 mL·min^−1^	6–11 mL·min^−1^	100% water	-	The hollow fiber module was placed horizontally.	Median particle size was 1.6–11.7 µm	[29]
[A] 2-Propanol; [CS] L-asparagine in de-ionized water.	0.175, 0.235 and 0.325 m·s^−1^.	50% of CS velocity; which means 0.087, 0.115 and 0.162 m·s^−1^	100% 2-propanol	-	The hollow fiber module appears to be placed vertically on the schematic.	Cross flow velocity 100 to 500 µm·s^−1^ Median crystal size was c.a. 70 µm	[22]
[A] Ethanol; [CS] Erythritol in water.	20 mL·min^−1^	60–120 mL·min^−1^	100% ethanol	-	The hollow fiber module was placed at angles 33, 50 and 55°.	Flux was 1.2–2.8 Kg·m^−2^·h^−1^; CV was 50.9%	[23]
[A] Ethanol; [CS] Glycine in water	0.00017–0.0005 m/s	0.00017–0.0005 m/s	40–100 wt.% ethanol	298.15–308.15	The solution circulation path was either through the upper or lower membrane cell.	Flux was 0.0002–0.001 Kg/m^2^·s; CV was 50–60% Mean particle size 23–40 µm	This study

## 2. Materials and Methods

### 2.1. Material

The α-Glycine was purchased from Sigma-Aldrich (Overijse, Belgium) and used as received in preparing the crystallizing solutions with a saturation concentration of 23 wt.%. Flat sheet Polypropylene membrane was purchased from 3 M GmbH (Neuss, Germany) and used as received. The membrane hydrophobicity was measured to be 150° and the SEM imaging of the cross section showed a sponge-like structure of the membrane (Figure S1, Supplementary Materials).

### 2.2. Membrane Performance for MAAC

**Figure 1:**
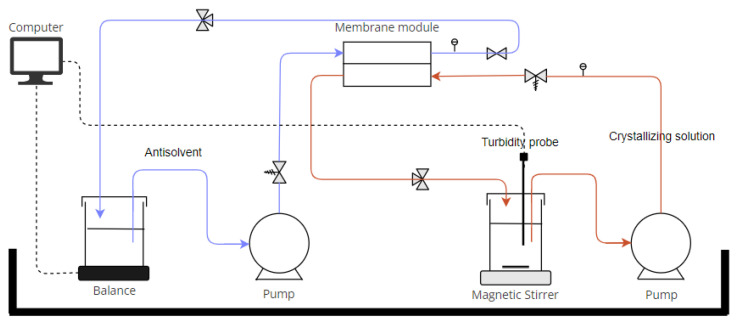
Schematic of the setup developed to evaluate the membrane performance of antisolvent crystallization under different conditions (variation of solutions velocity, antisolvent composition, temperature and gravity resistance).

The schematic in Figure 1 below and the real image in Figure S2 of Supplementary Materials illustrate the experimental set-up that was used to evaluate the membrane performance in MAAC. The crystallizing solution containing ultrapure water plus the dissolved solute was directed by a gear pump (Cole Parmer, Antwerp, Belgium) to the bottom cell of the membrane module. Likewise, the anti-solvent was circulated using the gear pump (Verder, Aartselaar, Belgium) in the top cell of the membrane module (DeltaE Srl, Lauria, Italy). 

As the anti-solvent permeated through the membrane contactor, its mass in the reservoir decreased, which was tracked by the collected data from the balance. On the other hand, the formation of crystals was tracked with the turbidity meter, indicating the beginning of the time crystallization is initiated. Glycine-ethanol-water was the model crystallizing system chosen for this study as it gives a neutral pH environment for the crystallization (so it cancels out the impact of pH) and has been generally used as a model molecule for antisolvent crystallization [30]. The variation of operating conditions is summarized in Table 2. Velocities of crystallizing and antisolvent solutions were computed from the flow rate *Q* delivered by the pumps using the following equation:

$$v=\frac{Q}{\pi r^{2}}$$

such that *v*, the solution velocity is in m/s, *Q* is in m^3^/s and *r*, the inlet radius in m.

#### 2.2.1. Antisolvent Transmembrane Flux Transport

The analytical balance allowed for the continuous tracking of the change of the antisolvent mass as it continued to permeate through the membrane pores to the crystallizing bulk solution. The weight values were then used to calculate the antisolvent transmembrane flux as follows:

(1) $$J=\frac{1}{A}\frac{w_{as}\left(t_{i}\right)-w_{as}\left(t_{i+1}\right)}{t_{i}-t_{i+1}}$$

with *J* the antisolvent transmembrane flux in kg/m^2^·s, *A* the active membrane area which was 0.012 m^2^, *w_as_* the weight of the antisolvent in kg and *t_i_* the operation time in seconds.

#### 2.2.2. Gas Chromatography (GC) for the Quantitative Analysis of Ethanol

Ethanol content was quantified using GC (Thermo Scientific™—TRACE 1300 Gas Chromatograph, Brussel, Belgium) equipped with a flame ionization detector (FID). Samples were retrieved at 10, 20, 40, 60, 80, 100 and 120 min of the experimental operation time, from both the crystallizing solution side and the antisolvent side. The former allows us to monitor the progress of ethanol content in the solution, responsible for inducing an increase in supersaturation, while the latter allows us to detect if there was any membrane wetting throughout the operation. An amount of 0.5 g of the retrieved sample was mixed with 0.5 g of acetonitrile solution. The exact mass was measured when preparing each sample to be sure of the accuracy of the results given by GC. The GC analysis was run at least twice for each sample, and a detailed explanation of its calibration was given in Supplementary Materials (Figure S3).

### 2.3. Crystal Quality

#### 2.3.1. Crystal Shape

The particle size and shape of the active pharmaceutical ingredients (APIs) are crucial quality attributes that are known to influence not only downstream processing such as filtration and drying but also the formulation processes and drug product attributes such as the rate of drug release and bioavailability [31,32,33]. All crystals were recovered from the bulk crystallizing solution at the end of each experiment, unless indicated otherwise. They were dried under the fume hood overnight, then grounded before prepping them for SEM imaging (Ultra 55 Feg Sem, Zeiss, Zaventem, Belgium). A thin layer of gold was deposited under vacuum with a sputter coater Quorum Q150 RS to make the samples conductive. The aspect ratio (AR) of the crystals (length/width) was evaluated using ImageJ tools and results are reported in Table S2a–f.

#### 2.3.2. Crystalline Form

To evaluate the crystallinity of glycine crystals, samples of crystals resulting from the membrane surface and the bulk solution were evaluated using X-ray diffraction (D8 Advance, Bruker, Kontich, Belgium) using Cu Kα radiation, λ = 1.5406 Å, in the range from 10 to 100 degrees.

#### 2.3.3. Crystal Size Distribution

The particle size distribution analysis was carried out using the SYNC particle analyzer (Microtrac MRB, Haan, Germany), which allows to examine materials over a wide range of sizes from 0.01 to 4000 microns, using static light scattering. This method is based on the deflection of a laser beam by an ensemble of particles dispersed in the liquid stream, and the angles of diffraction or scattering angles are characteristic of the particle size. A 150 W sonicator for 2 min was used to break the aggregates in 20–30 mL isopropanol. Figure S3 (Supplementary Materials) illustrates the impact of using sonication prior to analysis to avoid data distortion. A refractive index of 1.4264 was set for glycine. The camera measures 30,000 particles. The results reported by SYNC 5000 for data acquisition in this work are the average value of three performed measurements. 

#### 2.3.4. In-Line Turbidity Measurement

InPro 8200 turbidity meter (ELSCOLAB NV, Kruibeke, Belgium) was used to track the change of solution turbidity during the operation time of experiments. The in-line measurement allowed us to track the turbidity values at each second, which resulted in accurate quantification of the crystallization time.

## 3. Results and Discussion

### 3.1. Impact of Solution Velocity

**Figure 2:**
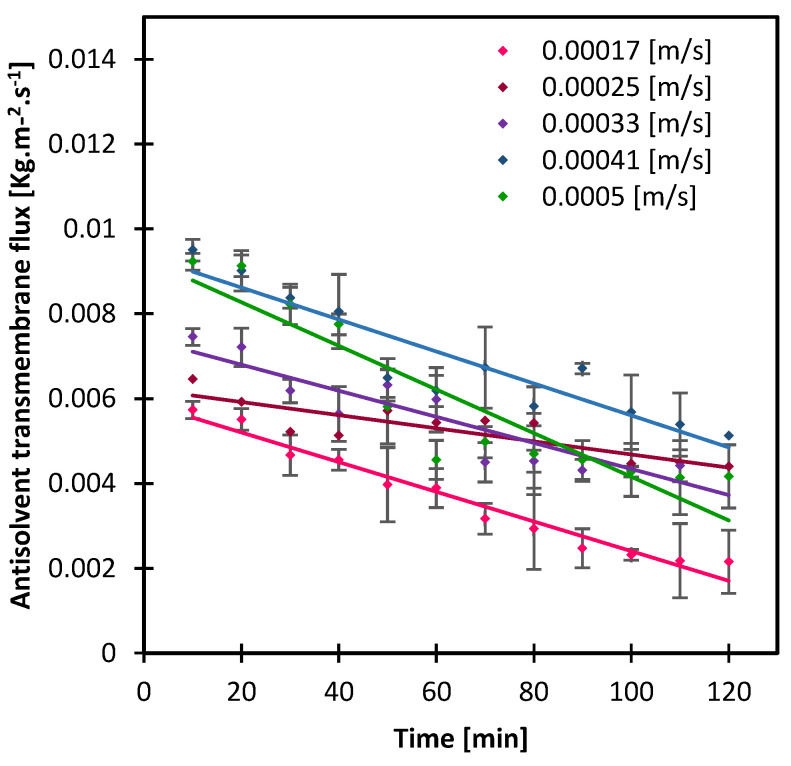
The mass transfer of the antisolvent through PP membrane as both the crystallizing and the antisolvent solutions, equally increased from 0.00017 to 0.0005 m/s. Lines are added to guide the eye.

To evaluate the impact of either the solution velocity or the crystallizing solution velocity, it was necessary to evaluate first the antisolvent mass transfer with the incremental of both solutions’ speed simultaneously and identify the most stable antisolvent transmembrane flux (Figure 2). The driving force to the mass transfer of the antisolvent through the membrane is related to different factors on top of which the concentration difference that is used in computing transmembrane flux. The variation of the solution velocity changes the hydrodynamic pressure of the solution from either side of the membrane, which alters the antisolvent addition rate as well. Notably, as the velocity of solutions increases, the thickness of liquid boundary layer at the membrane surface should be decreasing, which facilitates the transmembrane flux [28,34].

The rate of the transmembrane flux was the most stable at 0.00025 m/s, then gradually increased as the velocity of both streams increased simultaneously (Figure 2). The flux decline and the slight fluctuations that were observed can be attributed to the composition variation of the crystallizing solution with time, i.e., the rate at which the crystals appear and fill the solution, combined with the hydrodynamics of the solution. The higher the velocity of solution, the more shear is applied at the membrane surface which helps wash off crystals that form at the surface. Besides, as the antisolvent permeates to the crystallizing solution, the activity difference declines with time which also justifies the decline in transmembrane flux. Based on this, the impact of the crystallizing solution velocity while fixing the antisolvent at 0.00025 m/s and vice versa was next evaluated by looking at the stability of mass transfer and the resulting crystals retrieved from the solution.

**Figure 3:**
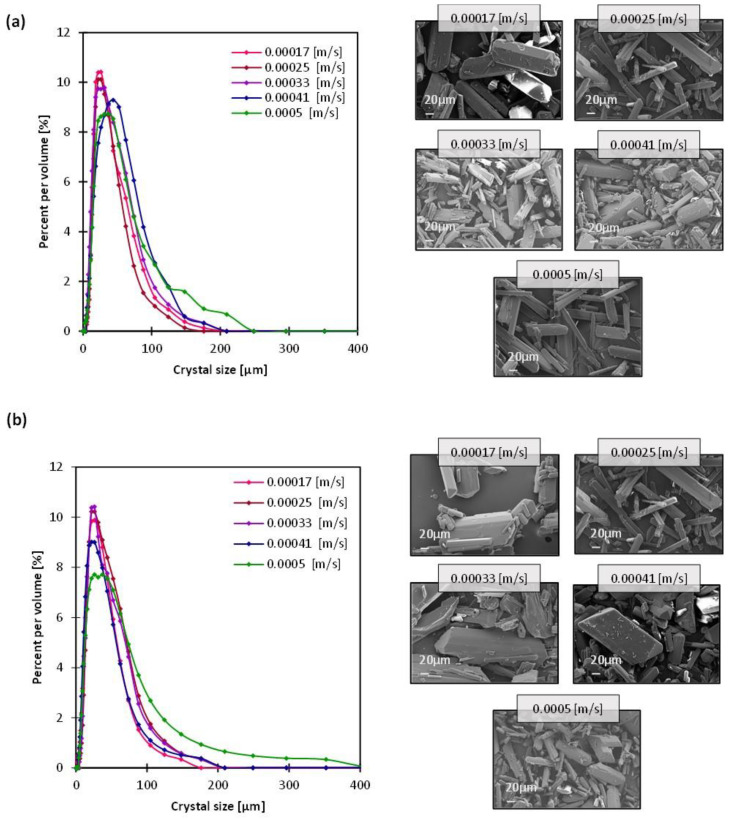
SEM images of crystals obtained from MAAC operation and the corresponding crystal size distribution, at incremental velocities of (**a**) the crystallizing solution and (**b**) the antisolvent solution.

**Figure 4:**
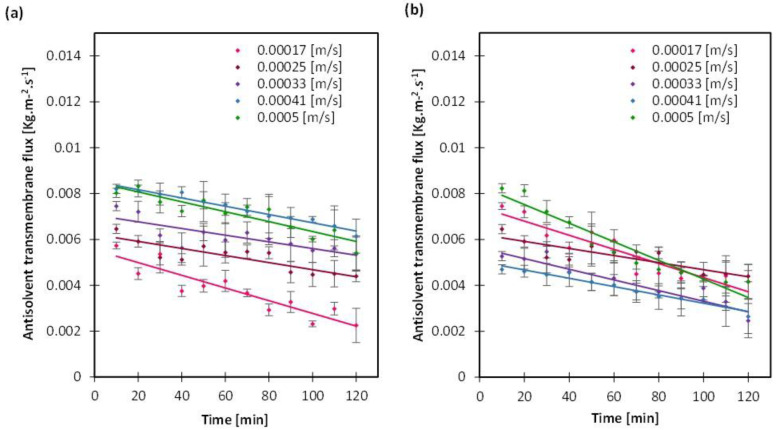
Antisolvent transmembrane flux with variation of (**a**) the crystallizing solution and (**b**) the antisolvent solution from 0.00017 to 0.00025 m/s. Lines are added to guide the eye.

In all cases of stream velocity variations, the crystal size distribution (CSD) was narrow whether the crystallizing solution or the antisolvent solution velocities increased (Figure 3). An optimum operation of MAAC requires both a minimum coefficient of variation (CV)—calculated from the CSD—besides a stable flux rate that enables a controlled antisolvent permeation. The CV values varied between 0.52 to 0.65 (Table S2a,b) which was lower than 0.7 that obtained with batch or drop-by-drop crystallization (Table S2f). Minimum CV values of 0.52 and 0.57 were obtained when the antisolvent solution was circulating at velocities of 0.00033 and 0.00041 m/s, respectively, (while the crystallizing solution was fixed at 0.00025 m/s). The same observation was made for the aspect ratio CV, which did not follow a clear trend either under the different conditions, but was in all cases lower than the resulting aspect ratio CV of either drop-by-drop or batch crystallization, reflecting the capacity of MAAC in maintaining the crystal morphology. Next, looking into the most stable transmembrane flux rate (Figure 4), the case where the antisolvent solution was set at 0.00041 m/s was eventually considered the most optimum MAAC operation, given the first factor—the velocity of streams.

In some previous studies, the impact of the velocity of either stream was not established, although seemingly some optimization must have taken place to ensure no membrane wetting and no counter permeation [24,28,29]. On the other hand, few other studies discussed this factor, either the flow rate of the crystallizing or the antisolvent solution. Tuo et al. [20] varied the crystallizing solution flow rate between 10–20 mL/min and the antisolvent solution between 60–120 mL/min, which suggested that MAAC operation is more sensitive to the flow rate of the crystallizing solution, impacting both the pressure at the membrane cell, and the mixing of solution. On the other hand, the variation of antisolvent’s velocity played a role in varying the boundary layer and hydraulic pressure, which impacted eventually the antisolvent permeation. This agrees with our findings although the mechanism applied here relied on the pressure difference between the two sides of the membrane contactor rather than the activity difference, by pushing the antisolvent in the liquid form through the pores, which explains why the resulting crystals here did not have a CSD as narrow as the one obtained from the current work. 

As for the impact of the membrane’s pore size, Fern et al. [29] varied the crystallizing solution flow rate between 10 and 20 mL/min using membranes of various pore sizes, and the mean crystal size increased as the crystallizing solution flow rate increased. No explanation was given regarding this correlation, but it agrees with the current findings and can be attributed to the effect of high mixing established by the use of the membranes. Zarkadas et al. [22] conducted mixing experiments with only water on one side of the membrane (being the solvent of the crystallizing solution), and the antisolvent solution flowing on the other membrane side, to evaluate the impact of solution velocity on the transmembrane mass transfer. When varying the solvent velocity from 0.175 to 0.325 m/s, higher antisolvent permeation was observed, predicting a higher rate of supersaturation when the actual API solution was to be tested. Finally, Li et al. [26] further confirmed the same correlation between the transmembrane antisolvent flux and the variation of either stream’ velocity owing to the variation of the liquid layer at the membrane vicinity, which was also demonstrated in their work using CFD simulations.

The mean crystal size varied between 27 and 40 µm, both the minimum and maximum values appeared when the antisolvent solution was set at 0.00017 and 0.0005 m/s while the crystallizing solution was fixed at 0.00025 m/s. There is no clear correlation between the mean crystal size and the streams velocities, although a difference was observed in the antisolvent transmembrane flux (Figure 4). This is apparent as well in the crystallization times (Figure S5a,b) that ranged between 83 and 100 min without a clear trend, whether as the crystallizing or the antisolvent solution circulation rates increased. In principle, it can be deduced that with the variation of the antisolvent solution velocity or the crystallizing solution velocity, one can operate in accurate conditions that allow for a controlled mass transfer and mixing of the crystallizing solution while avoiding membrane wetting. It is possible then to control crystallization by forming a narrow CSD and to avoid agglomeration while maintaining the crystal shape and purity, as was confirmed with the XRD spectra in Figure S6a,b.

### 3.2. Impact of Antisolvent Composition

**Figure 5:**
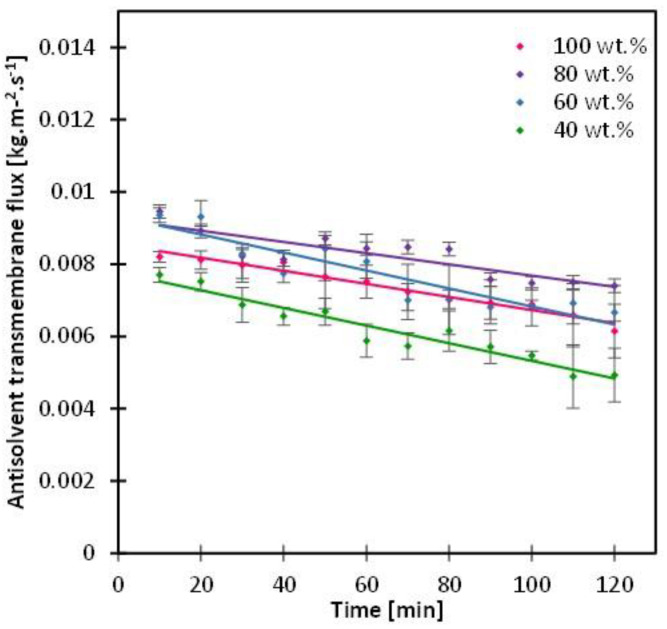
Antisolvent transmembrane flux with variation of antisolvent composition using an ethanol-water mixture of 100-80-60 and 40 wt.% ethanol. Lines are added to guide the eye.

The impact of antisolvent composition was investigated by reducing ethanol (the antisolvent) concentration in ethanol-water mixture with 80, 60 and 40 wt.%, using the mixture as the antisolvent solution instead of pure ethanol. All previous studies in the literature used 100% antisolvent solution [20,21,22,23,28,29] except Di Profio et al. [24], who used 40% ethanol in their system besides maintaining a temperature difference between the two sides of the membrane, to avoid membrane wetting by the crystallizing solution. The intent of reducing the concentration of the antisolvent is in theory to reduce the activity difference, hence it can further control the transmembrane flux rate; in other words, the antisolvent addition rate. In this study, indeed at 40 wt.%, the transmembrane flux was significantly lower than it was for 100 wt.% ethanol; on the other hand, the transmembrane flux was slightly higher (than it was for 100 wt.% ethanol) in the cases of 60 and 80 wt.% (Figure 5). This may appear counter-intuitive at first, but it can be attributed to the change in the membrane structure or the surface structure at specific water-ethanol compositions [35].

**Figure 6:**
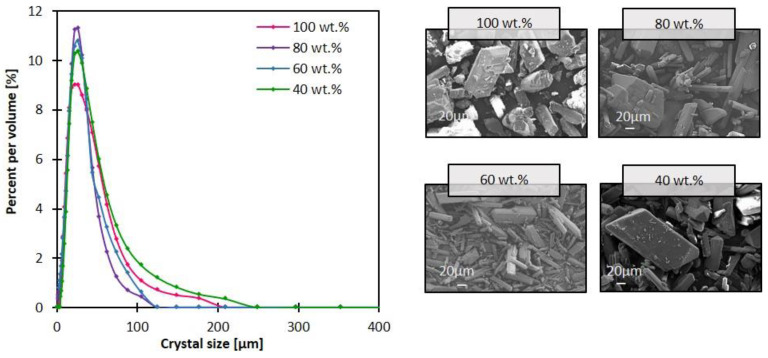
SEM images of crystals recovered from the bulk solution after the 2-h MAAC operation, and the corresponding crystal size distribution, with variation of antisolvent mixture.

The antisolvent transmembrane flux correlated directly with the resulting CSD such that an increase in mass transfer resulted in a narrower CSD (Figure 6), with CV values around 0.57 in all cases (Table S2c), and crystallization time ranging from 92 to 120 min (Figure S5b). The most uniform crystal morphology was obtained with 40 wt.% ethanol concentration with an aspect ratio CV of 28% compared with 32, 34 and 50% obtained by 60, 100 and 80 wt.% ethanol compositions. Regardless of the antisolvent composition, the crystal shape was intact and maintained its prism-like shape. This reckons that the crystal shape is indeed primarily related to the internal crystal habit plus the type of antisolvent used, which was in this study ethanol [36].

### 3.3. Impact of Temperature

**Figure 7:**
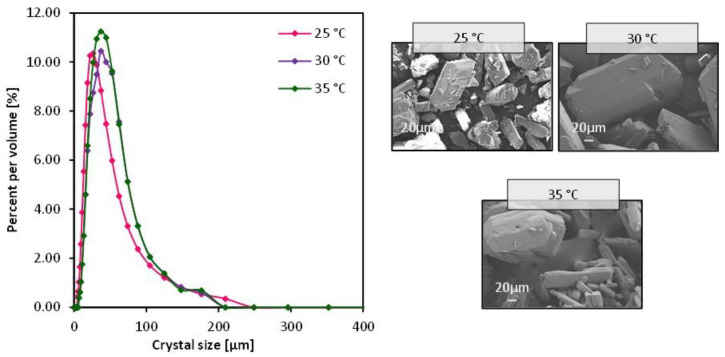
SEM images of crystals obtained from the bulk solution after MAAC operation and the corresponding crystal size distribution, with variation of temperature from 25 to 35 °C.

**Figure 8:**
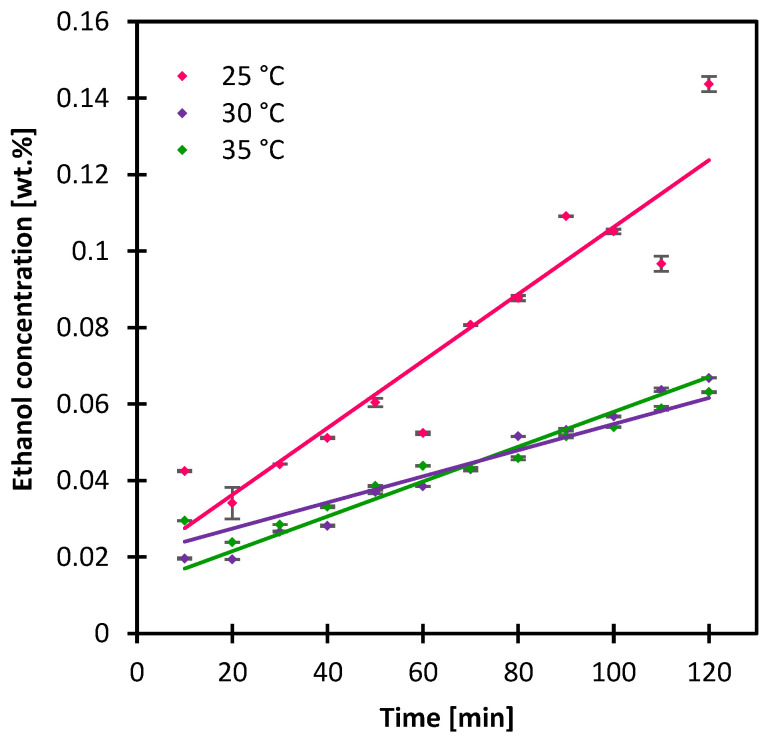
Ethanol concentration in the crystallizing solution at the first 120 min, with variation of operating temperature. Lines are added to guide the eye.

The impact of the operating temperature was evaluated by warming both the crystallizing and the antisolvent solution from 25 to 35 °C. The crystallization and the antisolvent solutions circulated at 0.00025 and 0.00041 m/s, respectively. It is worth noting that going beyond 35 °C did not result in any crystallization even after 12 h. Crystals appeared only after the solution reached room temperature after the experiment with the appearance of large aggregates. Based on this, no further increase in temperature was evaluated. In theory, temperature decreases the solubility of glycine, and can potentially impact the crystal size [26,36]. At 30 °C, it took more than twice the time it was necessary to see crystals with the initial state of 25 °C, and about an hour extra when the temperature was set at 35 °C (Figure S5d). Not much difference was observed in the CSD (Figure 7), with the CV values ranging from 0.54 to 0.58, indicating the narrowest CSD in the case where the temperature was set at 35 °C, and a most uniform crystal morphology at 30 °C with an aspect ratio CV of 29% (Table S2d). The mean crystal size on the other hand increased from 33, 34 to 37 µm with incremental of temperature from 25, 30 to 35 °C, respectively. In principle, crystal nucleation is inversely proportional to temperature [36], which means the higher the temperature, the more inhibited the nucleation mechanism versus crystal growth. Plus, obtaining smaller crystals imply the promotion of nucleation rather than crystal growth and vice versa [24]. Though crystal growth and nucleation rates were not quantified in this study, the induction time plus the crystal size variation could insinuate which phenomenon could have been promoted under each condition. The promotion of crystal growth was further reinforced with the slower permeation of the antisolvent through the membrane which was significantly lower in the cases of 30 and 35 °C compared with T = 25 °C (Figure 8).

As far as the crystal morphology is concerned, the crystals resulting from an operating temperature of 35 °C showed softer edges and surfaces; however, no apparent change was observed in the crystalline system of the resulting crystals (Figure S6d).

### 3.4. Impact of Gravity

**Figure 9:**
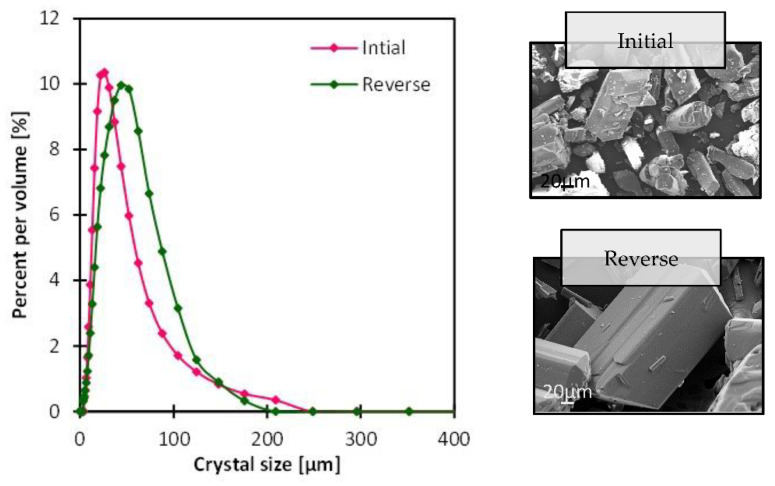
SEM images of crystals recovered from the bulk solution after MAAC operation and the corresponding crystal size distribution, with the variation of the gravity resistance.

**Figure 10:**
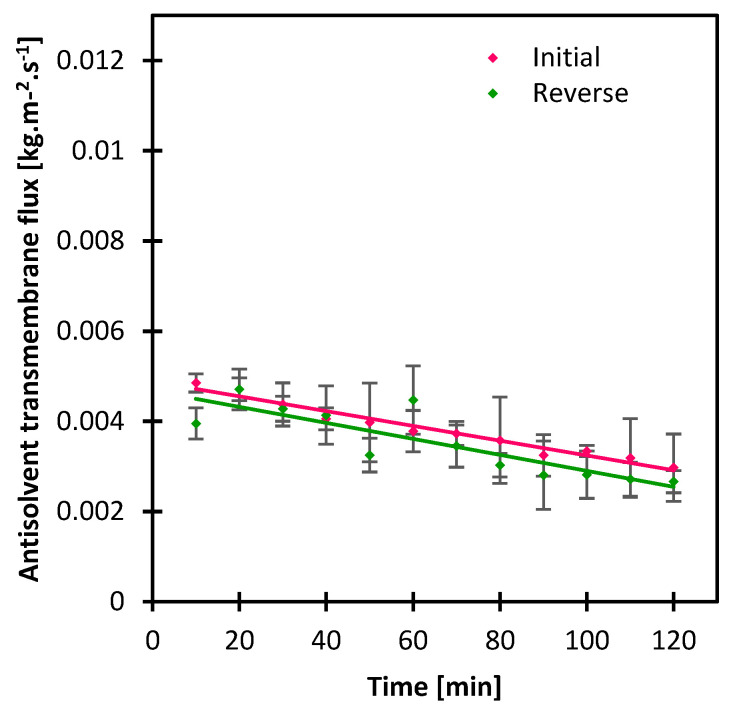
Antisolvent transmembrane flux with variation of gravity impact.

Gravity is a factor that was pointed out in the literature in several occasions for a proper adjustment of MAAC operation, in the sense that the membrane module can be put for example in a vertical position to prevent the crystal clogging inside the module or the tubing [20,21,22,28,29]. Li et al. put the membrane module at different angles of 33, 50 and 55° which impacted the density of crystals such that the bulk density decreased as the repose angle increased [23]. This shows that the module position impacts the forces applied by gravity on the antisolvent transmembrane mass transfer, which eventually influences the crystal formation kinetics. In this study, the impact of gravity was investigated by changing the position at which the antisolvent solution was circulating initially, and moved to the lower membrane cell instead, reinforcing the gravity resistance. The crystallizing solution velocity was kept at 0.00025 m/s and the antisolvent one at 0.00041 m/s, while the antisolvent composition was 40 wt.% ethanol. The crystal shape was intact (Figure 9) and more uniform with less gravity resistance given the aspect ratio CV was 12% lower than that obtained with the reverse condition. The crystal size distribution was narrow in either case with CV values of 0.57 and 0.61 in the initial and reverse condition, respectively (Table S2e). The transmembrane flux was slightly lower in the reverse condition (Figure 10) which can be attributed to the additional mass transfer resistance induced by gravity, and which reflected slightly on the crystallization time with 4 min difference (Figure S5e).

In all, the controlled transport of the antisolvent through the membrane pores resulted in a slow addition of the antisolvent to the crystallizing solution, and their homogeneous mixing as far as MAAC operated in the adequate range of either the solutions velocity, the antisolvent composition, or the temperature. The use of the membrane as the physical barrier between the crystallizing and the antisolvent solution avoids the burst of crystallization emerging from the local supersaturation at the antisolvent addition point whether in batch or drop-by-drop crystallization, which eventually gives a wide CSD (Figure S7). Gaining a deeper insight into the crystallization behavior at the membrane surface would be recommended for future work, especially since some residual crystals were observed at the membrane surface after MAAC operation and the crystals recovered from the surface had a larger size (Figures S8 and S9, Supplementary Materials). This gives preliminary clues on the role of the membrane as a substrate for heterogeneous crystallization allowing a crystal formation-growth-detachment mechanism. Smaller crystals detach perhaps easier to the bulk solution while larger ones remain in the membrane vicinity owing to the hydrodynamics difference between the membrane surface and the bulk solution. On an industrial scale, a membrane-based crystallizer would be good to avoid blending limitations that are faced by a conventional one. A proper membrane module analysis would be necessary for a successful scale-up maintaining the fluid dynamics. A close similarity between the transmembrane mass transfer of a bench and a scaled-up system should be expected, which would be key to attaining the same crystallization yield. 

## 4. Conclusions

This work presents the key factors that impact the operation of membrane-assisted antisolvent crystallization (MAAC). The objective of this work was to identify the effect of (i) the velocity of either the crystallizing or the antisolvent solution, (ii) the antisolvent composition, (iii) the operating temperature and (iv) the influence of gravity.

The present study together with the previous studies agreed that an increase in the crystallizing solution velocity reduces the mass transfer resistance, helps wash off crystals forming at the membrane surface and provides greater interfacial mixing between the system solvent-antisolvent-solute. On the other hand, an increase in the antisolvent solution velocity reduces the thin film layer formed at the membrane surface which avoids wetting and promotes the antisolvent transmembrane flux. There was not a particular trend observed with either the mean crystal size or the coefficient of variation (reflecting the quality of crystal distribution). CV values ranged from 0.52 to 0.65, while the mean crystal sizes ranged from 27 to 40 µm. The case where the antisolvent was set at 0.00041 m/s was chosen as the most optimum, having the most stable transmembrane flux rate and one of the lowest CV values.

As far as the antisolvent composition is concerned, the antisolvent transmembrane flux reached its lowest at 40 wt.% ethanol with the CV value of 0.57 and the mean crystal size of 33 µm, which was the largest compared to the cases of 60, 80 and 100 wt.% ethanol reflecting promotion of growth mechanism rather than nucleation, as the permeation of the antisolvent was much slower in this case. This reflected the greater control of the antisolvent through the membrane; hence, 40 wt.% ethanol was considered the most optimum condition.

As for the temperature, a clear trend was observed with the mean crystal size such that it increased at the temperature increased from 25 to 35 °C, and the CV value was the lowest at the latter condition. The rate of ethanol permeation was much lower at 30 and 35 °C compared with the initial state of 25 °C. However, the 35 °C temperature slightly impacted the resulting crystal morphology such that the crystal surfaces and edges appeared smoother than in any previous case where the crystal edges appeared perfectly sharp. Finally, the gravity impacted slightly the transmembrane flux resulting in crystals of a larger mean crystal size with seven microns and a 4% difference in the CV of the crystal size distribution. It is then recommended to operate with a configuration that minimizes the impact of gravity as was the case in previous studies, placing the module vertically for better flowability and avoiding crystal clogging.

In brief, MAAC could successfully elevate the problem of instant crystallization that is observed with batch or drop-by-drop crystallization where the CV of crystal distribution reached 0.7 at best. It is important to consider parameters that are in favor of controlling the antisolvent mass transfer through the membrane while providing optimum mixing in the crystallizing solution, which is the condition where the antisolvent velocity is slightly higher than that of crystallizing solution, the antisolvent composition is either 100 or 40 wt.% depending on the intended crystal size (the latter gives larger crystals); finally, operating at ambient temperature (depending on the crystal solubility) and placing the module in a position limiting gravity resistance, would be much recommended.

## Figures and Tables

**Table 2:** Operating conditions used in the current study of MAAC.

Parameter	Variable
Solution velocity, either the crystallizing solution or the antisolvent.	0.00017, 0.00025, 0.00033, 0.00041 and 0.0005 m/s
Antisolvent composition	40, 60, 80 and 100 wt.%
Temperature	25, 30 and 35 °C
Gravity	Antisolvent circulating in the upper or the lower membrane cell.

## Data Availability

The data presented in this study are available on request from the corresponding author.

## Supplementary Materials

The supporting information can be downloaded at: https://www.mdpi.com/article/10.3390/membranes13020140/s1. Figure S1: SEM cross-sectional image of polypropylene (PP) membrane; Figure S2: Real image of MAAC set-up; Figure S3: Effect of sonication prior to CSD analysis; Figure S4: The calibration curve of GC; Figure S5a–e: The turbidity measurement of the crystallizing solution during all MAAC experiments; Figure S6a–e: The XRD spectra of Glycine crystals recovered from the bulk solution after MAAC experiments; Figure S7: The crystal size distribution of the commercial glycine (as received) and the crystals recovered from batch and drop by drop crystallization.; Figure S8: SEM surface image of PP membranes before and after 2-h operation of MAAC; Figure S9: SEM images of crystals recovered from the bulk crystallizing solution and the membrane surface after 2-h operation of MAAC, while the crystallizing and antisolvent solution velocities varied simultaneously; Table S1: List of chemicals used in this study; Table S2a–f: Crystal size and aspect ratio.
