# Supplementary material for: Key Parameters Impacting the Crystal Formation in Antisolvent Membrane-Assisted Crystallization
Source: Membranes (Basel). 2023 Jan 21;13(2):140. doi: 10.3390/membranes13020140 (PMC9964214; doi:10.3390/membranes13020140)
Supplement: Supplementary file 1 [file membranes-13-00140-s001.zip › membranes-2132965-supplementary.pdf]

Supplementary Materials

Key parameters impacting the crystal formation antisolvent membrane-assisted crystallization

Sara Chergaoui[1,2*], Damien P. Debecker[3], Tom Leyssens[3], and Patricia Luis[1,2*]

[1] Institute of Mechanics, Materials and Civil Engineering—Materials & Process Engineering (iMMC-IMAP), UCLouvain, Place Sainte Barbe 2, 1348 Louvain-la-Neuve, Belgium
[2] Research & Innovation Centre for Process Engineering (ReCIPE), UCLouvain, Place Sainte Barbe, 2 bte L5.02.02-B, 1348 Louvain-la-Neuve, Belgium
[3] Institute of Condensed Matter and Nanosciences (IMCN), UCLouvain, Place Louis Pasteur, 1 bte L4.01.06, 1348 Louvain-la-Neuve, Belgium; tom.leyssens@uclouvain.be (T.L.); damien.debecker@uclouvain.be (D.P.D.)

* Correspondence: sara.chergaoui@uclouvain.be (S.C.); patricia.luis@uclouvain.be (P.L.)

List of chemicals

Table S1: List of chemicals used in this study.

Chemical	Characteristics	Supplier
Ultrapure Water	18.2 MΩcm	Lab
Glycine	**CAS:** 56-40-6	Sigma-Aldrich
Ethanol absolute	**CAS:** 64-17-5 **MW:** 46.07	VWR Chemicals

Image of Polypropylene membrane

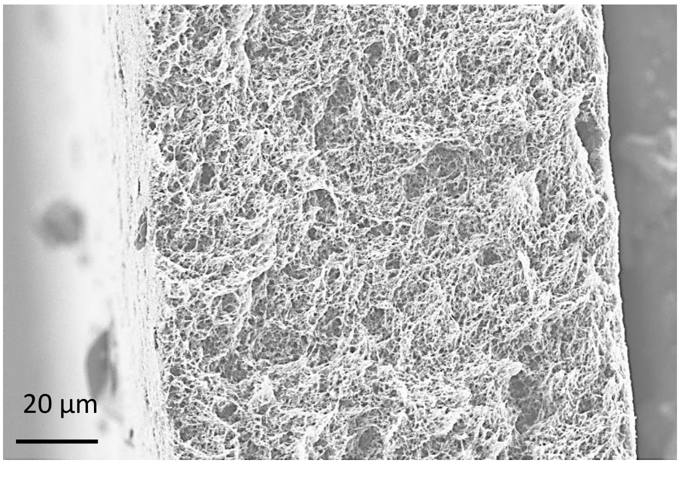

Figure S1: SEM cross-sectional image of polypropylene (PP) membrane showing a sponge-like structure.

Real image of Experimental set-up

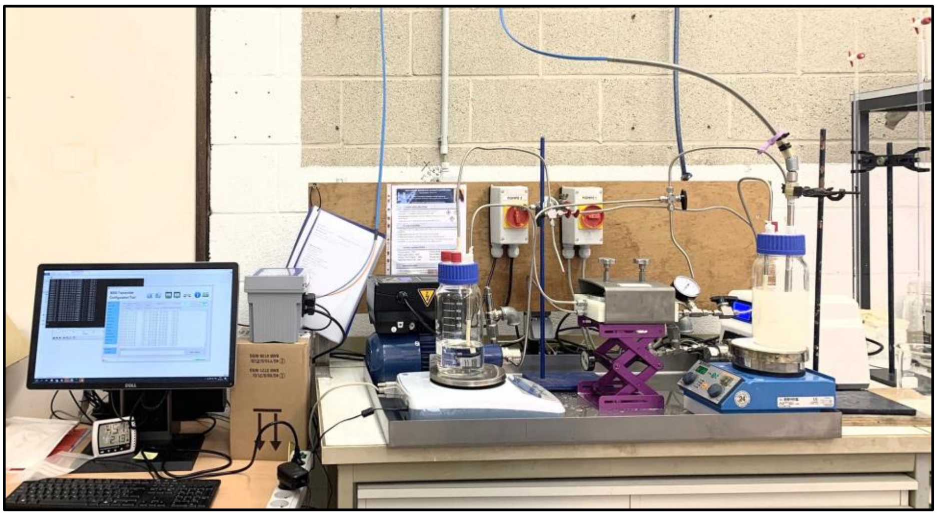

Figure S2: Real image of membrane-assisted antisolvent crystallization set-up. The crystallizing solution and the antisolvent are flowing in cross-flow mode in each side of the membrane module. The membrane module possessed a membrane area of 0.012 m^2. The turbidity probe tracks the appearance of crystal solids inside the feed solution while the balance keeps track of the variation of the antisolvent balance throughout the experiments.

Impact of sonication

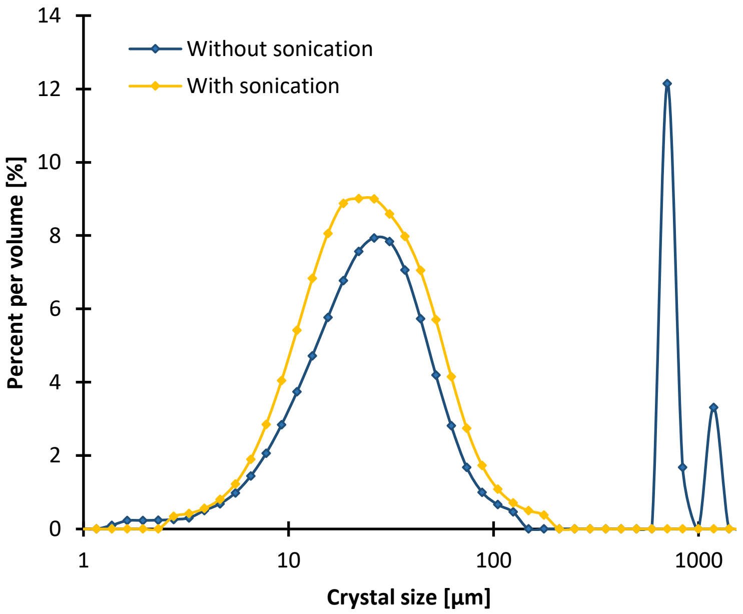

Figure S3: A 2-minute sonication was used prior to CSD analysis to avoid presence of aggregates in the sample that distorts the data distribution.

Calibration curve of GC

GC was calibrated using acetonitrile solution as a standard solution, mixed with ethanol solutions of known concentrations. Ethanol 1% (w/v), was mixed with 1% (w/v) acetonitrile at various ratios (ethanol: acetonitrile = 15:1, 10:1, 5:1, 2:1, 1:1, 1:2, 1:5, 1:10, and 1:15). A linear regression line was generated with the ethanol to acetonitrile concentration ratio (Y-axis) versus area-under-curve (AUC) ratio given by GC (X-axis). The resulting calibration curve is shown in (Figure S4).

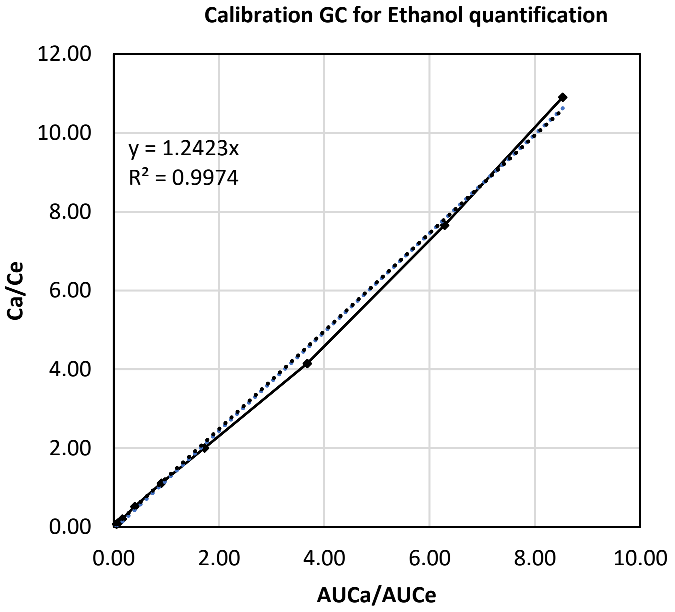

Figure S4: The calibration curve of GC.

Crystal size and aspect ratio

Table S2-a: Mean crystal size and coefficient of variation (CV) of the recovered crystals as the crystallizing solution velocity increased.

	0.00017 m/s	*0.00025 m/s*	*0.00033 m/s*	*0.00041 m/s*	*0.0005 m/s*
SD	17.57	19.75	16.23	23.55	23.52
Mean	29.05	34.26	27.76	38.08	38.76
Size CV	0.60	0.58	0.58	0.62	0.61
Aspect ratio CV	0.56	0.39	0.65	0.38	0.40

Table S2-b: Mean crystal size and coefficient of variation (CV) of the recovered crystals as the antisolvent solution velocity increased.

	0.00017 m/s	*0.00025 m/s*	*0.00033 m/s*	*0.00041 m/s*	*0.0005 m/s*
SD	16.5	19.75	18.28	16.54	26.16
Mean	27.78	34.26	35.02	28.79	40.23
Size CV	0.59	0.58	0.52	0.57	0.65
Aspect ratio CV	0.27	0.39	0.22	0.34	0.34

Table S2-c: Mean crystal size and coefficient of variation (CV) of the recovered crystals as the antisolvent concentration in Ethanol/water mixture decreased.

	100 wt.%	*80 wt.%*	*60 wt.%*	*40 wt.%*
SD	16.54	13.12	14.17	18.95
Mean	28.79	22.60	24.07	33.35
Size CV	0.57	0.58	0.59	0.57
**Aspect ratio *CV***	0.34	0.50	0.32	0.28

Table S2-d: Mean crystal size and coefficient of variation (CV) of the recovered crystals as the temperature increased.

	25 °C	*30 °C*	*35 °C*
SD	18.95	20.45	20.07
Mean	33.35	35.04	37.00
Size CV	0.57	0.58	0.54
**Aspect ratio *CV***	0.34	0.29	0.41

Table S2-e: Mean crystal size and coefficient of variation (CV) of the recovered crystals as the gravity resistance increased.

	Initial	*Reverse*
SD	18.95	24.52
Mean	33.35	40.21
Size CV	0.57	0.61
**Aspect ratio *CV***	0.34	0.46

Table S2-f: Mean crystal size and coefficient of variation (CV) of the commercial Glycine, and that recovered from batch and drop-by-drop crystallization.

	Commercial Glycine	*Batch*	*Drop by drop*
SD	83.26	77.52	73.36
Mean	105.50	103.40	101.2
Size CV	0.79	0.75	0.72
**Aspect ratio *CV***	0.52	0.48	0.43

Turbidity

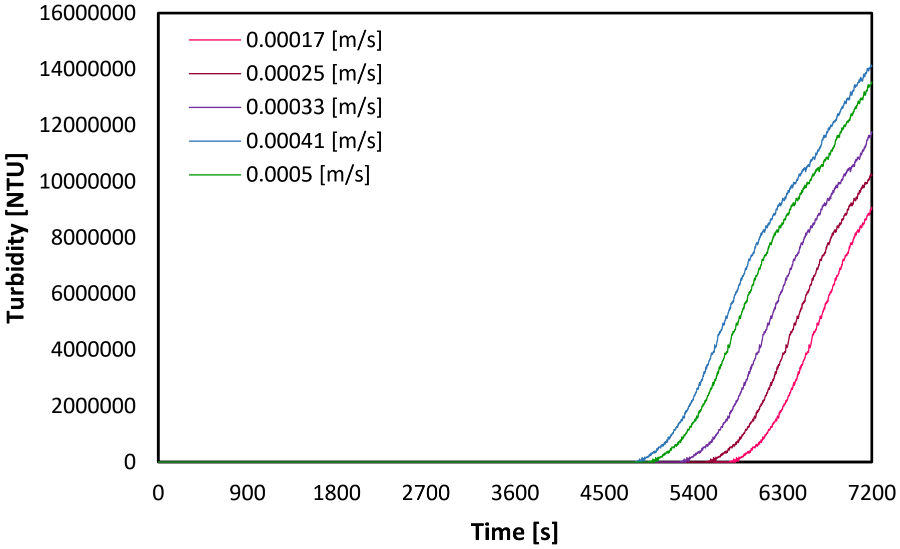

Figure S5-a: The turbidity measurement of α-Glycine solution as the velocity of the crystallizing solution increases. The crystallization times are 90, 94, 92, 100 and 84 min when the crystallization solutions were set at 0.00017, 0.00025, 0.00033, 0.00041 and 0.0005 m/s.

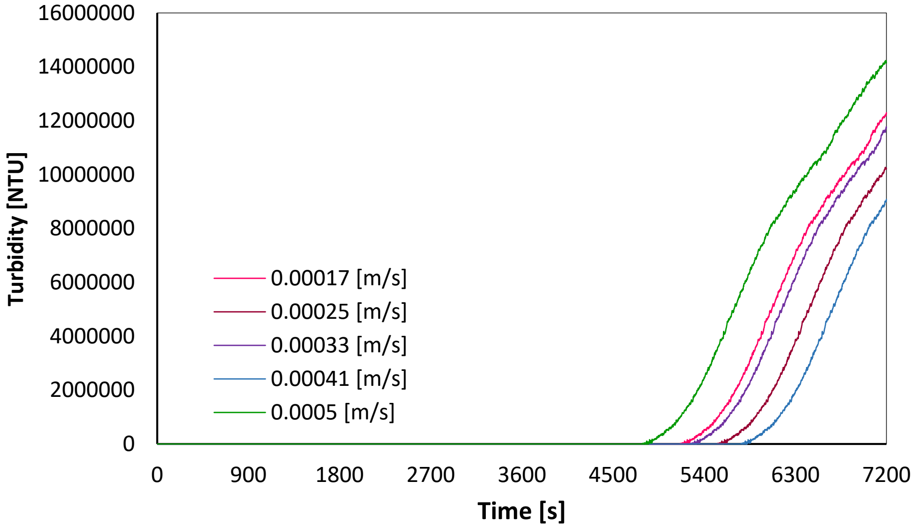

Figure S5-b: The turbidity measurement of α-Glycine solution as the velocity of the antisolvent solution increases. The crystallization times are 99, 94, 90, 84 and 83 min when the crystallization solutions were set at 0.00017, 0.00025, 0.00033, 0.00041 and 0.0005 m/s.

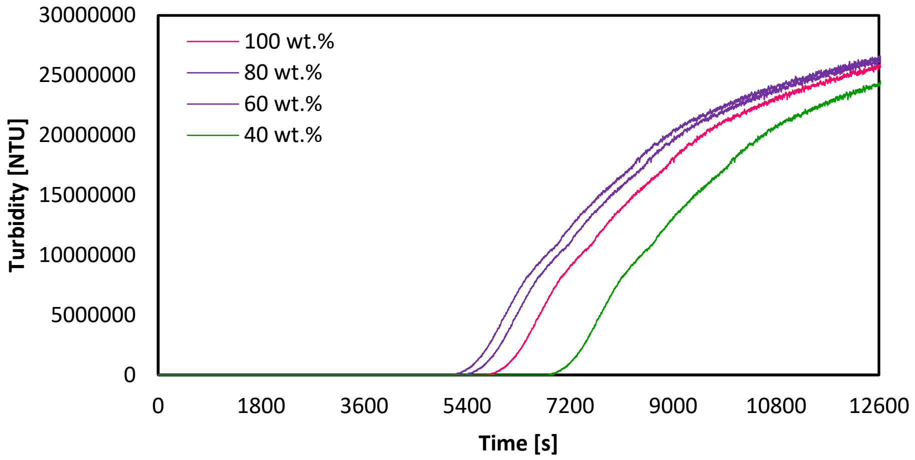

Figure S5-c: The turbidity measurement of α-Glycine solution as the velocity of the crystallizing solution increases. The crystallization times are 102, 90, 92 and 120 min when the crystallization solutions were set at 0.00017, 0.00025, 0.00033, 0.00041 and 0.0005 m/s.

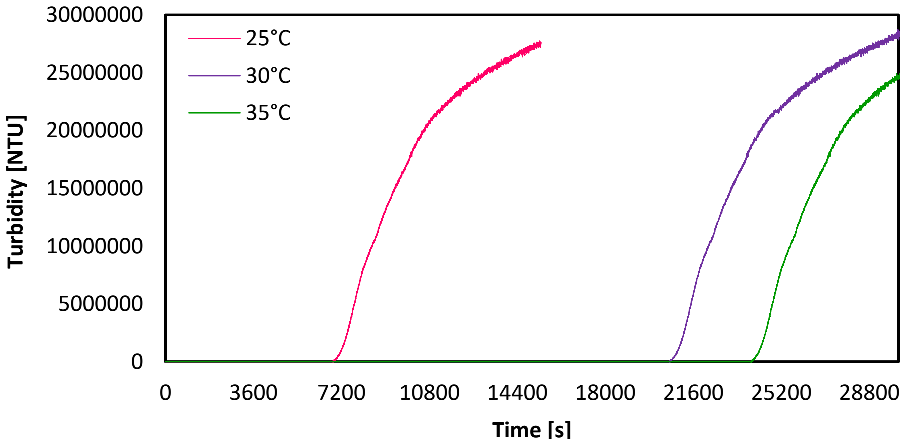

Figure S5-d: The turbidity measurement of α-Glycine solution as the velocity of the crystallizing solution increases. The crystallization times are 120, 351 and 400 min when the crystallization solutions were set at 0.00017, 0.00025, 0.00033, 0.00041 and 0.0005 m/s.

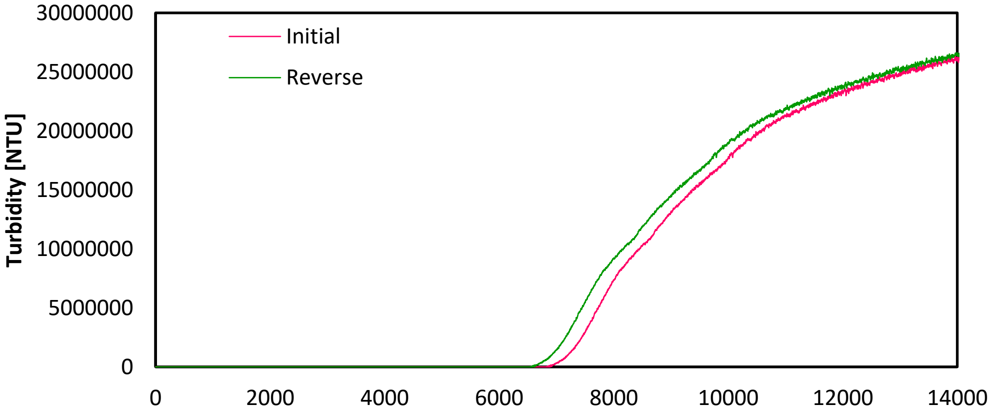

Figure S5-e: The turbidity measurement of α-Glycine solution as the velocity of the crystallizing solution increases. The crystallization times are 116 and 120 min for the initial and reverse configuration where more gravity resistance is taking place. The crystallizing and antisolvent solutions were set at 0.00017 and 0.0005 m/s respectively.

Crystallinity

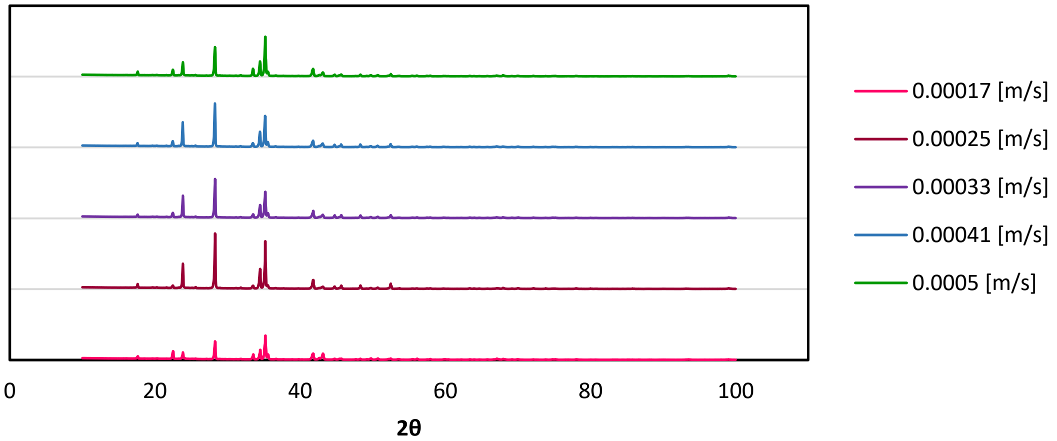

Figure S6-a: The XRD spectra of Glycine crystals recovered from the bulk solution after the 2-hour MAAC experiment, with incremental of crystallizing solution velocity.

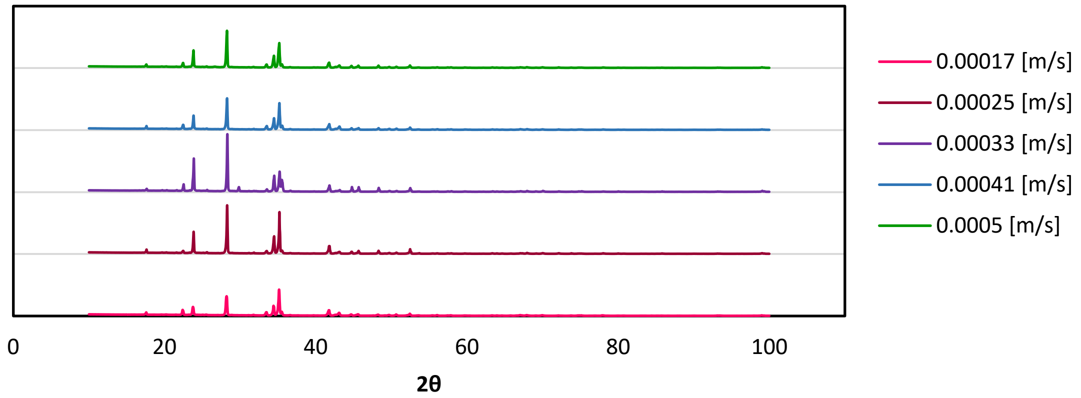

Figure S6-b: The XRD spectra of Glycine crystals recovered from the bulk solution after the 2-hour MAAC experiment with incremental of antisolvent solution velocity.

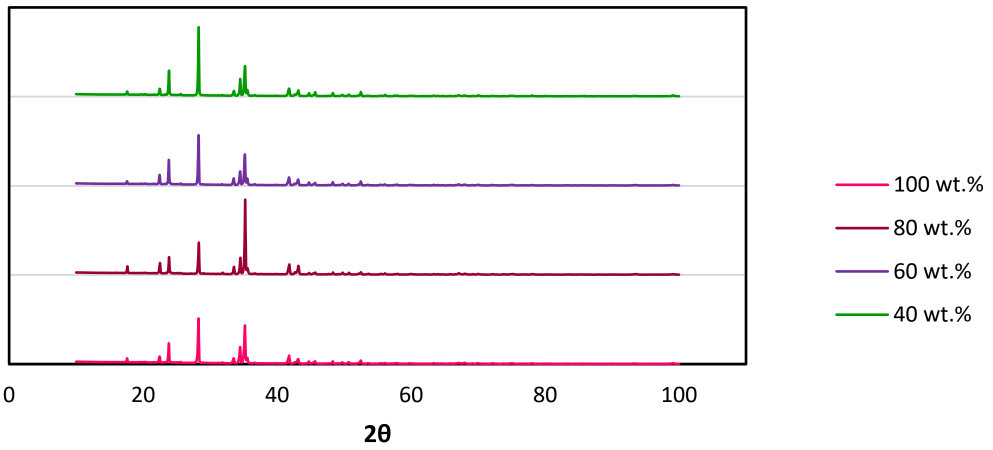

Figure S6-c: The XRD spectra of Glycine crystals recovered from the bulk solution after the 2-hour MAAC experiment, with variation of antisolvent composition.

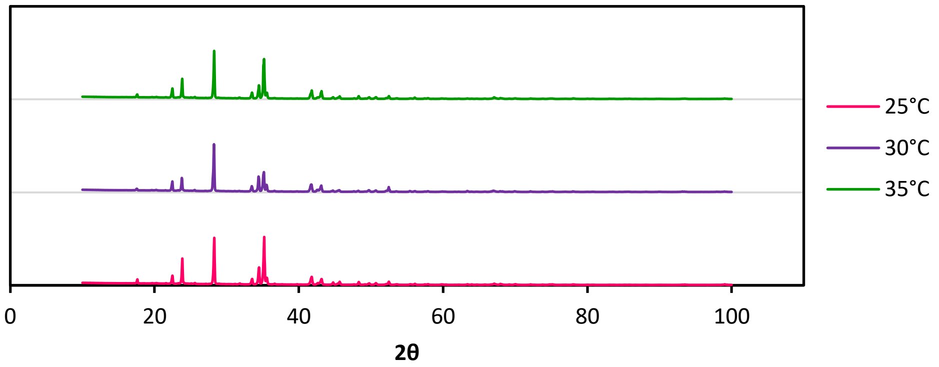

Figure S6-d: The XRD spectra of Glycine crystals recovered from the bulk solution after the 2-hour MAAC experiment with variation of temperature.

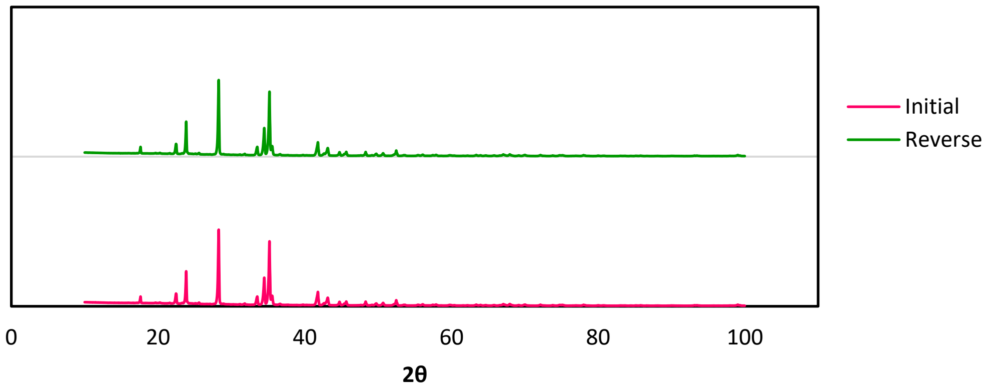

Figure S6-e: The XRD spectra of Glycine crystals recovered from the bulk solution after the 2-hour MAAC experiment with variation of gravity resistance.

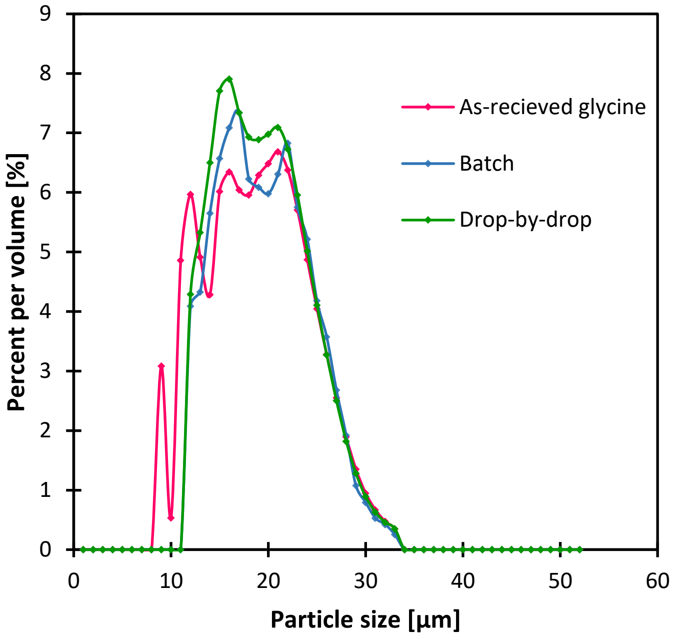

Figure S7: The crystal size distribution of the commercial glycine (as-received) and the crystals recovered from batch and drop-by-drop crystallization.

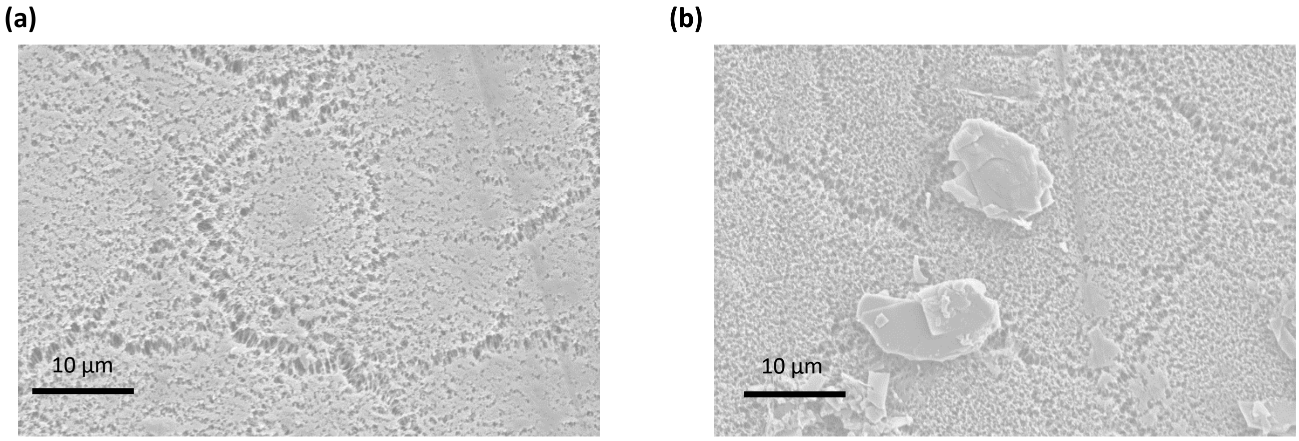

Figure S8: SEM surface image of PP membranes before and after 2-hour operation of MAAC when the crystallizing and antisolvent solutions circulated at 0.00025 and 0.00041 m/s respectively. The residual crystals left at the surface indicate the importance of the solution's turbulence to wash off crystals from the vicinity of the surface.

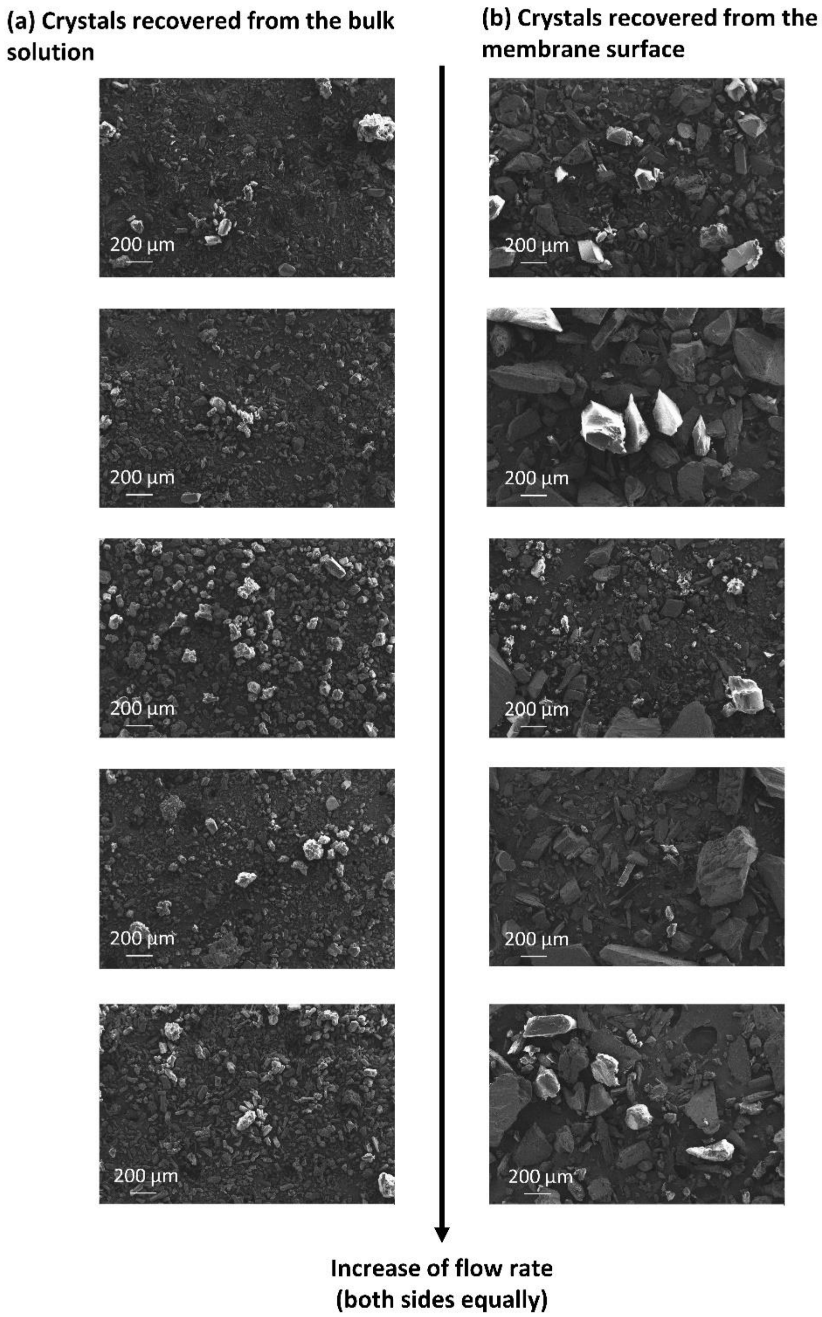

Figure S9: SEM images of crystals recovered from the bulk crystallizing solution and the membrane surface after 2-hour operation of MAAC. The crystallizing and antisolvent solution velocities varied simultaneously from 0.00017, 0.00025, 0.00033, 0.00041 and 0.00025 m/s. In all, the crystals recovered from the membrane surface had a larger size than those recovered from the bulk solution.
